# Nuclear mitochondrial DNA insertions are prevalent in the human brain and accumulate over time in fibroblasts

**DOI:** 10.1002/alz.085457

**Published:** 2025-01-03

**Authors:** Weichen Zhou, Kalpita R Karan, Wenjin Gu, Hans‐Ulrich Klein, Gabriel Sturm, Philip L. De Jager, David A. Bennett, Michio Hirano, Martin Picard, Ryan E Mills

**Affiliations:** ^1^ University of Michigan Medical School, ANN ARBOR, MI USA; ^2^ Columbia University Irving Medical Center, New York, NY USA; ^3^ University of Michigan Medical School, Ann Arbor, MI USA; ^4^ Center for Translational and Computational Neuroimmunology, New York, NY USA; ^5^ Rush Medical College, Chicago, IL USA

## Abstract

**Background:**

The transfer of mitochondrial DNA into the nuclear genomes of eukaryotes (Numts) has been linked to lifespan in non‐human species and recently demonstrated to occur in rare instances from one human generation to the next.

**Method:**

Here we investigated numtogenesis dynamics in humans in two ways. First, we quantified Numts in 1,187 post‐mortem brain and blood samples from different individuals. Second, we tested the dynamic transfer of Numts using a repeated‐measures whole genome sequencing design in a human fibroblast model that recapitulates several molecular hallmarks of aging.

**Result:**

In the ROSMAP dataset, compared to circulating immune cells (n = 389), post‐mitotic brain tissue (n = 798) contained more Numts, consistent with their potential somatic accumulation. Within brain samples we observed a 5.5‐fold enrichment of somatic Numt insertions in the dorsolateral prefrontal cortex compared to cerebellum samples, suggesting that brain Numts arose spontaneously during development or across the lifespan. Moreover, more brain Numts was linked to earlier mortality. The brains of individuals with no cognitive impairment who died at younger ages carried approximately 2 more Numts per decade of life lost than those who lived longer. In the lifespan model, the longitudinal experiments revealed a gradual accumulation of one Numt every ∼13 days. Numtogenesis was independent of large‐scale genomic instability and unlikely driven cell clonality. Targeted pharmacological perturbations including chronic glucocorticoid signaling or impairing mitochondrial oxidative phosphorylation (OxPhos) only modestly increased the rate of numtogenesis, whereas patient‐derived *SURF1*‐mutant cells exhibiting mtDNA instability accumulated Numts 4.7‐fold faster than healthy donors.

**Conclusion:**

Combined, our data document spontaneous numtogenesis in human cells and demonstrate an association between brain cortical somatic Numts and human lifespan. These findings open the possibility that mito‐nuclear horizontal gene transfer among human post‐mitotic tissues produces functionally‐relevant human Numts over timescales shorter than previously assumed.

